# First person – Jessica Sharrock

**DOI:** 10.1242/dmm.039776

**Published:** 2019-04-04

## Abstract

First Person is a series of interviews with the first authors of a selection of papers published in Disease Models & Mechanisms (DMM), helping early-career researchers promote themselves alongside their papers. Jessica Sharrock is first author on ‘*fs(1)h* controls metabolic and immune function and enhances survival via AKT and FOXO in *Drosophila*’, published in DMM. Jessica conducted the research described in this article while a PhD student in Marc S. Dionne's lab at MRC Centre for Molecular Bacteriology and Infection, Imperial College London, UK. She is now a postdoc in the lab of Joseph C. Sun at Immunology Program, Memorial Sloan Kettering Cancer Center (MSKCC), NY, USA, investigating the metabolic function of immune cells, particularly natural killer cells, during viral infection and cancer.


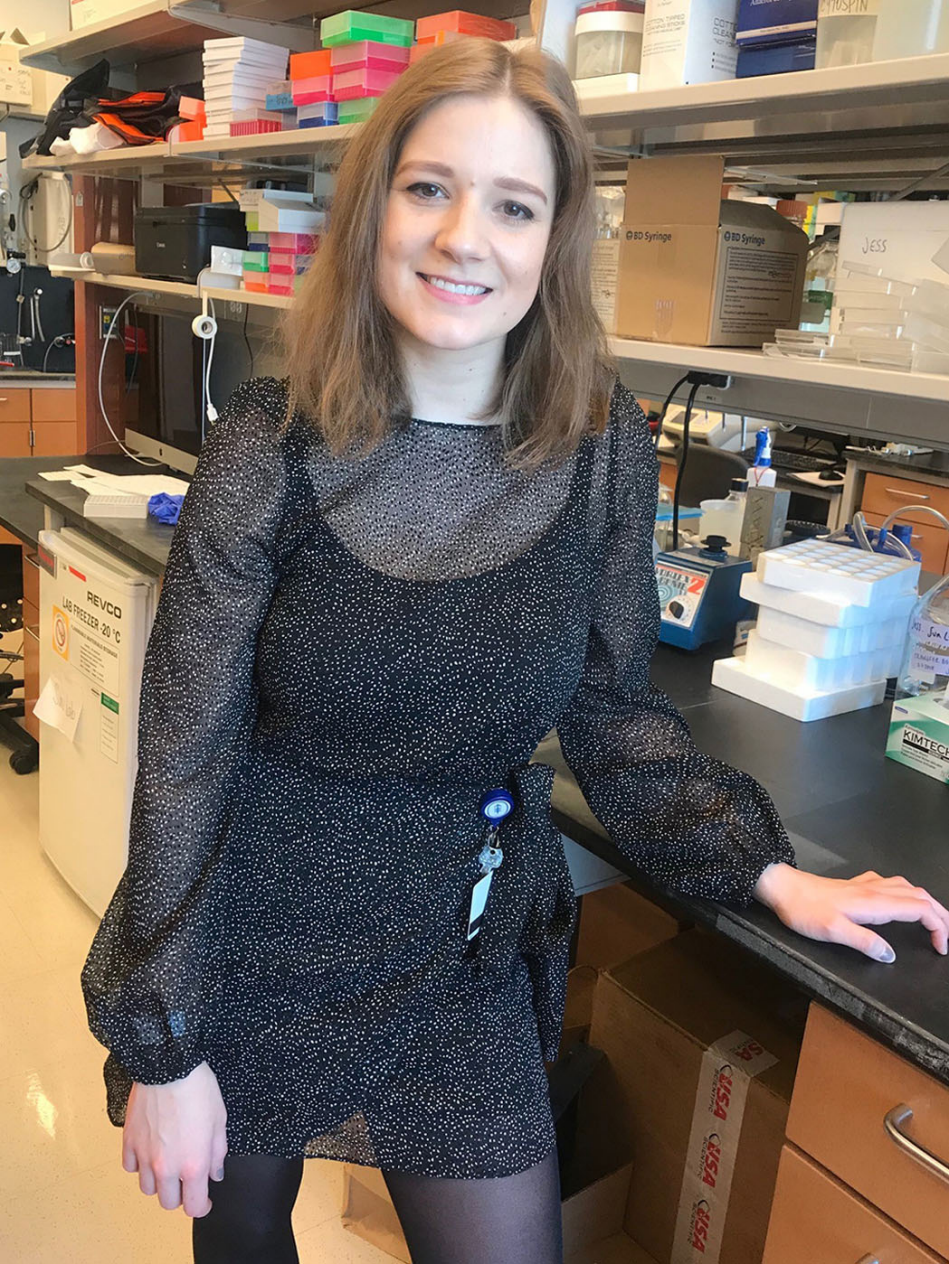


**Jessica Sharrock**

**How would you explain the main findings of your paper to non-scientific family and friends?**

The fat body of the fruit fly, *Drosophila melanogaster*, much like the mammalian liver and fat/adipose tissue, is the main site of energy storage and is crucial for the initiation of an immune response following infection. Our paper focuses on the *Drosophila* protein FS(1)H, a member of the evolutionarily conserved bromodomain-containing protein (BCP) family which helps regulate transcription, a process by which cells make a copy of their DNA, and remodel chromatin (a complex of DNA and proteins, which packs long DNA molecules into more compact structures). We found that genetically removing FS(1)H specifically from the fat body had detrimental effects on *Drosophila* survival, immune function and metabolic processes. Most of our findings resulted from failings in insulin signaling, a key pathway regulating the storage and uptake of glucose and lipids into the liver, adipose tissue and muscle. By additionally removing one copy of the gene *foxo*, a transcription factor (which helps turn specific genes ‘on’ or ‘off’ by binding to DNA) that can be regulated by the insulin pathway, in the flies lacking FS(1)H in the fat body, we were able to reverse many of the observations reported. Together, our work suggests that *fs(1)h* is important in the fat body for promoting insulin signaling activity in the whole organism.

“I hope that [our results] will also help to direct other interesting and exciting findings in other model organisms and in humans.”

**What are the potential implications of these results for your field of research?**

Bromodomains (BRDs) are evolutionarily conserved protein-protein interaction units found in a wide range of proteins. They are able to recognize and bind to acetylated lysine residues in histones, suggesting that they play essential roles in the regulation of gene expression. In recent years, it has become clear that BRDs are often dysregulated in cancer and have become important drug targets for many types of cancer. BRD inhibition is now an extremely significant area of research, and small-molecule inhibitors targeting the bromodomain and extraterminal domain (BET) family of BRDs are being tested in numerous clinical trials. However, in many contexts, little is known about their *in vivo* systemic functions. I hope that our results will begin to shed some light on the systemic function of BET proteins, not only in *Drosophila*: I hope that they will also help to direct other interesting and exciting findings in other model organisms and in humans.

**What are the main advantages and drawbacks of the model system you have used as it relates to the disease you are investigating?**

For over a century, *Drosophila* have proven to be a valuable genetic model to help understand fundamental biological processes. They have fast generation times, low maintenance costs, only four chromosomes and numerous genetic tools, making them a desirable model. However, there are still some limitations to the *Drosophila* system, including gene mutations not always being representative of human gene mutations (making translational science sometimes difficult) and the lack of some resources such as antibodies. However, for this study, the fly was a great model as FS(1)H is the sole BET protein, compared to mammalian models, which have four (BRD2, BRD3, BRD4, BRDT), removing some of the complexities of gene compensation and functional overlap.

**Figure:**
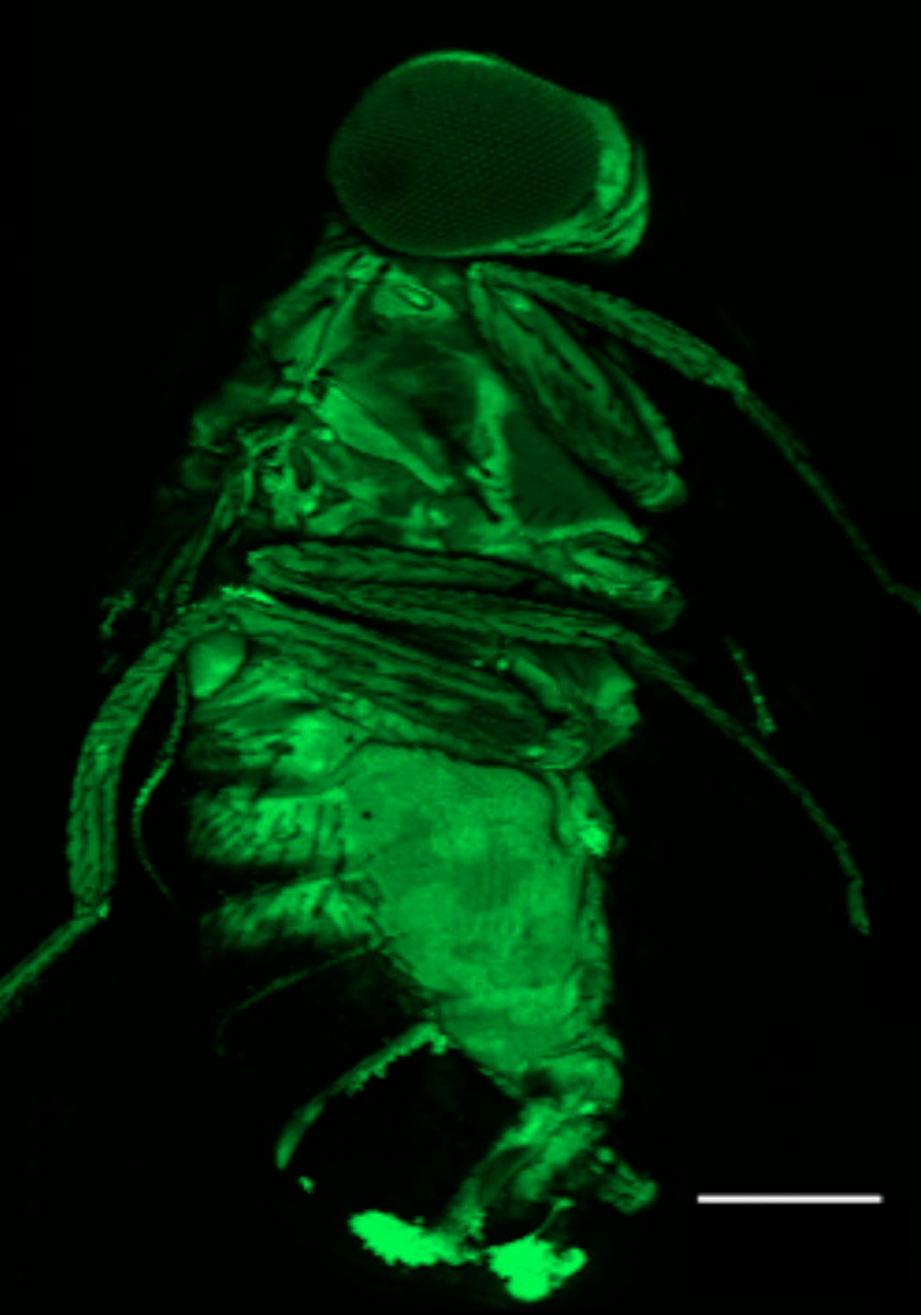
**The fruit fly, *Drosophila melanogaster*, imaged 1 h post-injection with fluorescently labelled 2-deoxy-2-D-glucose (2-NBDG), used to monitor glucose uptake in living cells. Scale bar: 300 µm.**

**What has surprised you the most while conducting your research?**

I think one of the biggest surprises for me was the incredibly vibrant and helpful *Drosophila* community, which was really beneficial as a PhD student. People from all over the world are willing to share resources, discuss ideas and provide constructive feedback. Additionally, I think until you work with *Drosophila* you can't comprehend the power and potential it has as a model organism. The development of such an array of genetic tools and techniques in such a small organism will continue to fascinate me.

 “I think one of the biggest surprises for me was the incredibly vibrant and helpful *Drosophila* community… People from all over the world are willing to share resources, discuss ideas and provide constructive feedback.”

**Describe what you think is the most significant challenge impacting your research at this time and how will this be addressed over the next 10 years?**

Being an early-career scientist and a female is a challenge impacting not only my research, but all the other women in a similar situation. Although opinions and circumstances within institutions are changing for the better, there is still a huge gender gap, particularly in immunology, within the more senior positions. I like to believe that, within the next 10 years, this is something we won't have to worry about or even think about.

**What changes do you think could improve the professional lives of early-career scientists?**

I think as a PhD student or a postdoc it's extremely important to have a supportive and inspiring mentor. As a postdoc I feel really lucky to have a mentor that is everything I need to grow and develop as an early-career scientist, but I know that that's not the case for everyone. Improvements in mentorship, leadership and supporting a healthy work-life balance at both the PhD and postdoc level is key for the next generation of scientists. Although it is difficult to achieve, increasing the funding opportunities for early-career scientists would enable considerable improvements to professional lives and hopefully remove some of the financial stress that can come with being an early-years scientist.

**What's next for you?**

I made the move from London to New York a little over a year ago for a postdoc position at MSKCC and it's been an incredible experience both scientifically and non-scientifically! For now, I'm very much focused on strengthening and broadening my immunology knowledge, continuing to work on metabolic signalling pathways in a more complex model system, and enjoying everything that New York has to offer.
